# A sulfur host based on titanium monoxide@carbon hollow spheres for advanced lithium–sulfur batteries

**DOI:** 10.1038/ncomms13065

**Published:** 2016-10-20

**Authors:** Zhen Li, Jintao Zhang, Buyuan Guan, Da Wang, Li-Min Liu, Xiong Wen (David) Lou

**Affiliations:** 1 School of Chemical and Biomedical Engineering, Nanyang Technological University, 62 Nanyang Drive, Singapore 637459, Singapore; 2 Beijing Computational Science Research Center, Beijing 100084, China; 3 State Key Laboratory of Silicon Materials, School of Materials Science and Engineering, Zhejiang University, Hangzhou, 320027, China

## Abstract

Lithium–sulfur batteries show advantages for next-generation electrical energy storage due to their high energy density and cost effectiveness. Enhancing the conductivity of the sulfur cathode and moderating the dissolution of lithium polysulfides are two key factors for the success of lithium–sulfur batteries. Here we report a sulfur host that overcomes both obstacles at once. With inherent metallic conductivity and strong adsorption capability for lithium-polysulfides, titanium monoxide@carbon hollow nanospheres can not only generate sufficient electrical contact to the insulating sulfur for high capacity, but also effectively confine lithium-polysulfides for prolonged cycle life. Additionally, the designed composite cathode further maximizes the lithium-polysulfide restriction capability by using the polar shells to prevent their outward diffusion, which avoids the need for chemically bonding all lithium-polysulfides on the surfaces of polar particles.

## Abstract (web summary)

The promise of lithium-sulfur batteries with higher energy densities than lithium-ion is hindered by the insulating nature of sulfur and dissolution of polysulfides. Here the authors design titanium monoxide/carbon hollow nanospheres that overcome both obstacles, enabling improved electrochemical properties.

Rechargeable battery systems are a vital part of many emerging applications, such as grid electrical storage and electric vehicles. Among existing electrochemical systems, lithium–sulfur (Li–S) batteries show advantages for next-generation electrical energy storage and conversion due to their high theoretical energy density, low cost and environmental friendliness^1^. However, the commercialization of the rechargeable Li–S battery is still hindered by three main issues of: (a) the inherent poor electronic conductivity of sulfur and its end products of discharge (Li_2_S/Li_2_S_2_), (b) the dissolution of intermediate lithium polysulfides (LiPSs), and (c) large volumetric expansion of ∼80% upon full lithiation. These issues bring about serious self-discharge, low Coulombic efficiency and rapid decline of capacity upon cycling^2^. Strenuous efforts have been devoted to improve the electrochemical performance of Li–S batteries, including composing sulfur with conductive materials^3^,^4^,^5^,^6^,^7^, constructing LiPSs blocking interlayers^8^,^9^,^10^,^11^, developing new electrolytes^12^,^13^,^14^ and applying functional binders^15^,^16^,^17^. Among these methods, the most popular strategy is composing sulfur with carbonaceous materials^6^,^7^,^18^,^19^, since their intrinsic good conductivity and diversity in nanostructures make the carbon materials very attractive^19^,^20^,^21^,^22^. However, the carbon/sulfur composite cathodes still generally suffer from rapid capacity fading over long-term cycling, because the nonpolar carbon can only provide weak physical adsorption to the polar LiPSs^23^. Once LiPSs are solvated, they can easily dissolve into the organic electrolyte from the electrode surface and diffuse away. Subsequent reutilization of LiPSs for capacity contribution will become very difficult due to the repulsion between the polar reactants and the nonpolar conductive surface^24^.

Recently, it has been realized that polar functional groups/surfaces can significantly increase the chemical interaction between polysulfides and the substrates^23^,^24^, and many efforts have been expended to develop sulfur hosts with strong chemical adsorption capability for LiPSs. For instance, heteroatom doping^25^,^26^,^27^ and surfaces functionalization^28^,^29^,^30^ of carbon materials lead to significant improvement of chemical adsorption of LiPSs. Taking advantage of the Lewis acid–base interactions with polysulfides, metal organic frameworks^31^, MXene nanosheets^32^ and metal hydroxides^33^,^34^ have been employed as sulfur hosts and achieved good cycling stability. Polar metal oxides/sulfides, such as SiO_2_ (ref. 35), TiO_2_ (ref. 36), indium tin oxide^37^, MnO_2_ (refs 38, 39), TiS_2_ (ref. 40) and CoS_2_ (ref. 41) can also adsorb polysulfides more tightly than carbon substrate, and provide significantly improved cycling properties. However, many metal oxides/sulfides usually have intrinsically poor electrical conductivity, thus the chemically adsorbed polysulfides are difficult to be reduced directly on the hosts' surfaces, resulting relatively lower sulfur utilization. To enhance the electrical conductivity of polar metal oxides/sulfides for improving the redox kinetics of the electrode, Ti_4_O_7_ nanoparticles^42^,^43^ and Co_9_S_8_ nanosheets^44^ have been employed as new concept sulfur hosts with both good conductivity and polar nature in one host. So far, utilization of polar materials with LiPSs anchoring abilities is viewed as an important strategy to confine polysulfide species and avoid their dissolution^24^. Yet the study of polar materials as sulfur hosts is still in its early stages. Most polar materials are applied just in the irregular particulate form, which can only adsorb LiPSs near the surfaces (Fig. 1a). This imposes another important issue one cannot avoid. Specifically, when the sulfur content is higher than a certain value, the polar material would not be able to provide sufficient interfaces to fix all of the LiPSs in the electrode (Fig. 1b). Therefore, more efficient LiPSs-trapping structures are in urgent need to solve the technical challenges of sulfur cathodes in Li–S batteries.

One practical way is to apply the least amount of polar materials as the shell of nanochambers (Fig. 1c). The polar hollow nanostructure has at least two main advantages: (i) the polar shell only needs to adsorb part of the LiPSs near the entrance, and then the internal deep-seated LiPSs will be naturally hindered from dissolution, (ii) the large void space of hollow materials not only allows loading of relatively higher content of sulfur, but also accommodates the large volumetric expansion of sulfur during lithiation. Beyond that, to obtain higher utilization of sulfur for higher capacities, the electrical conductivity of the polar hollow host also needs to be enhanced (Fig. 1d). Unfortunately, it is very challenging to synthesize metal oxides with both hollow nanostructure and high conductivity, due to the required high temperature annealing process for producing conductive metal oxides like Magnéli phase titanium oxides (Ti_*n*_O_2*n*−1_, *n*=4∼10) (ref. 45). Therefore, the application of hollow nanostructured conductive metal oxides as the sulfur host is rarely reported.

Herein, we design and synthesize polar hollow nanospheres with highly conductive shells constituted of titanium monoxide (TiO) and carbon as the sulfur host. With a high density of oxygen and titanium vacancies, the rock-salt structured stoichiometric TiO exhibits excellent electrical conductivity, which is nearly one order of magnitude higher than that of Magnéli phase Ti_*n*_O_2*n*−1_ (*n*=4∼10) (refs 45, 46), and its chemical resistance is similar to that of Ti_4_O_7_ (ref. 47). Density functional theory (DFT) calculations indicate that TiO can provide more effective adsorption of LiPSs than TiO_2_. Additionally, unlike previously reported granular sulfur hosts^42^,^43^, the designed titanium monoxide@carbon hollow spheres (TiO@C-HS) maximizes the LiPSs restriction capability by using just the polar shells to effectively prevent the outward diffusion of LiPSs, which breaks the limitation of chemically bonding all LiPSs on the surfaces of polar particles. As a result, the TiO@C-HS/S composite cathode delivers excellent comprehensive electrochemical properties even without using any other additive material.

## Results

### Materials synthesis and characterization

**Figure 2:**
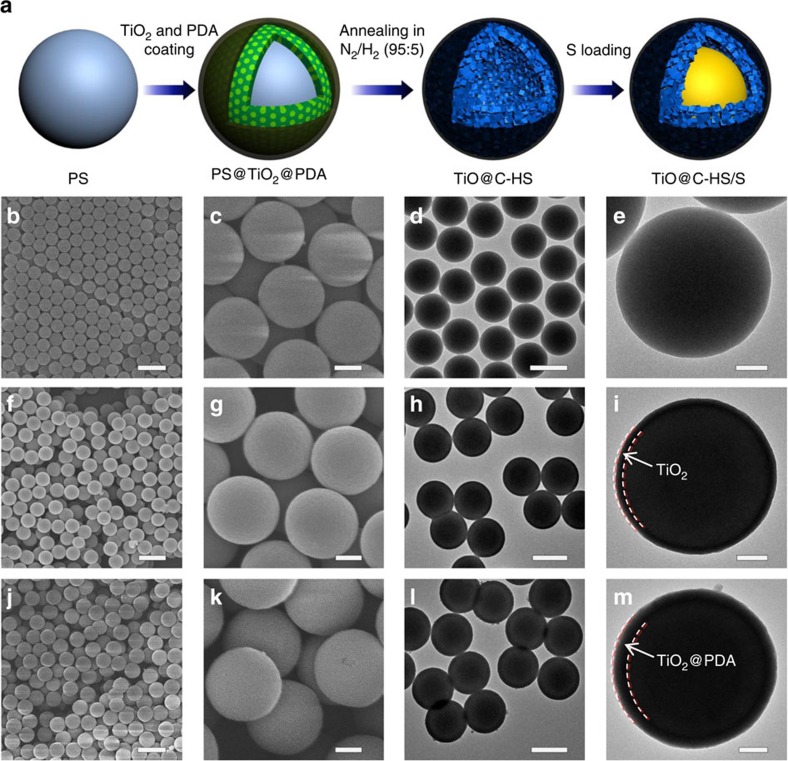
Synthesis process of the TiO@C-HS/S composite. (**a**) Schematic illustration of the synthesis process of the TiO@C-HS/S composite. SEM and TEM images of (**b**–**e**) PS spheres, (**f**–**i**) PS@TiO_2_ core-shell spheres and (**j**–**m**) PS@TiO_2_@PDA spheres. Scale bars, 1 μm (**b**,**f**,**j**), scale bars, 200 nm (**c**,**g**,**k**), scale bars, 500 nm (**d**,**h**,**l**), scale bars, 100 nm (**e**,**i**,**m**).

The synthesis strategy of the TiO@-HS/S composite is illustrated in Fig. 2a. Uniform polystyrene (PS) spheres are prepared as the template via a modified method reported elsewhere^48^. As shown in the scanning electron microscopy (SEM) and transmission electron microscope (TEM) images in Fig. 2b–e, the PS nanospheres are highly uniform with an average diameter of about 490 nm. Subsequently, a thin layer of amorphous TiO_2_ is uniformly coated on the surface of PS nanospheres through a cooperative assembly-directed strategy^49^. The PS@TiO_2_ core-shell structured nanospheres are also nearly monodisperse with an average diameter of about 550 nm (Fig. 2f–h), indicating that the thickness of the TiO_2_ shell is around 30 nm, in agreement with the TEM observation (Fig. 2i). After coated with a layer of polydopamine (PDA), the PS@TiO_2_@PDA sample still well maintains the spherical morphology with smooth surfaces (Fig. 2j–l), indicating PDA is uniformly polymerized outside the PS@TiO_2_ spheres. Since the thickness of the shell on PS is increased to ∼40 nm (Fig. 2m), the thickness of PDA layer is presumed to be about 10 nm.

**Figure 3:**
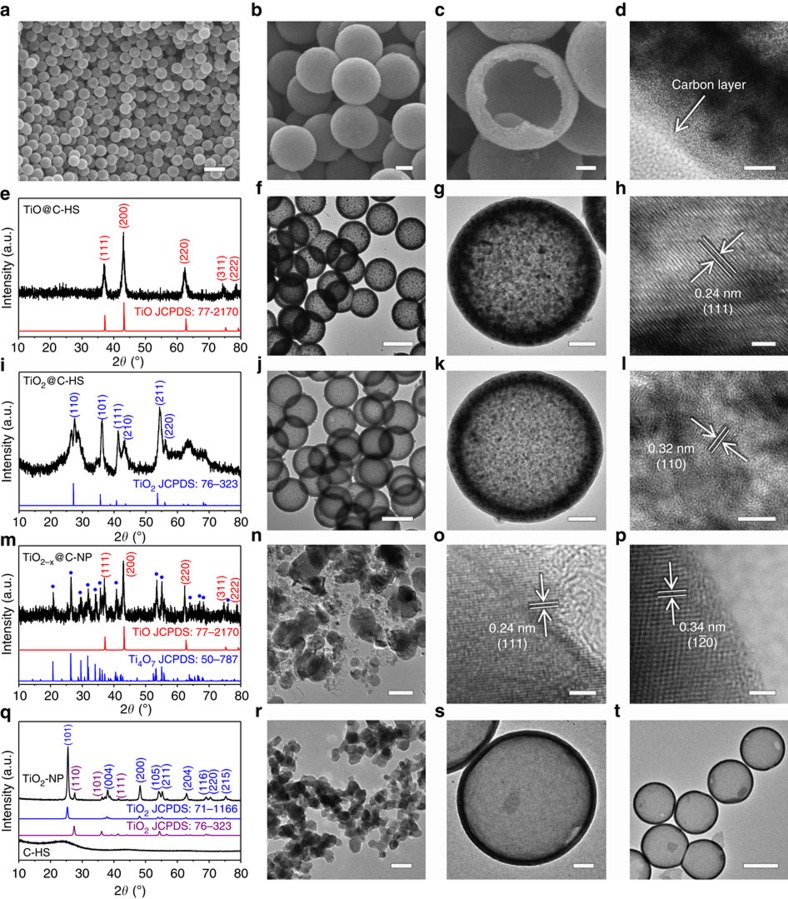
Characterization of TiO@C-HS and other control host materials. (**a**–**c**) SEM images, (**e**,**i**,**m**,**q**) X-ray diffraction patterns, (**d**,**f**–**h**,**j**–**l**,**n**–**p**,**r**–**t**) TEM images of (**a**–**h**) TiO@C-HS, (**i**–**l**) TiO_2_@C-HS, (**m**–**p**) TiO_2−*x*_@C-NP, (**q**,**r**) TiO_2_-NP and (**s**,**t**) C-HS. Scale bars, 1 μm (**a**), scale bars, 200 nm (**b**), scale bars, 100 nm (**c**,**g**,**k**,**n**,**s**), scale bars, 500 nm (**f**,**j**,**t**), scale bars, 10 nm (**d**), scale bars, 5 nm (**l**), scale bars, 2 nm (**h**,**o**,**p**), scale bars, 50 nm (**r**). a.u., arbitrary unit.

In order to transform TiO_2_ to conductive TiO, the PS@TiO_2_@PDA sample is annealed in a reductive atmosphere of N_2_/H_2_ (95:5) mixture gas at 1,000 °C for 4 h. TiO@C-HSs maintain uniform morphology with intact spherical shells, and the average diameter is ∼550 nm (Fig. 3a,b). The hollow structure of TiO@C-HSs can be identified from a broken one (Fig. 3c). The outer PDA layer is transformed into amorphous carbon after the annealing treatment (Fig. 3d). X-ray diffraction analysis (Fig. 3e) indicates that TiO (JCPDS No. 77-2170) is the primary crystalline phase of the product. TEM images show that the obtained TiO@C-HSs has uniform hollow architecture (Fig. 3f), and the inner shell is composed of abundant small nanocrystals (Fig. 3g). The clear lattice fringes with an inter-planar spacing of 0.24 nm can be readily assigned to the (111) planes of rock-salt TiO (Fig. 3h). It is interesting to note that, without the outer PDA layer, the PS@TiO_2_ core-shell particles will be transformed into large-sized irregular particles after the same high-temperature annealing treatment, and the spherical morphology is completely destroyed (Supplementary Fig. 1). Therefore, the presence of the protective outer carbon layer is very crucial for controlling the crystal phase and size of the inner TiO. Thermogravimetric analysis reveals that the carbon content in the TiO@C-HS structure is ∼43 wt% (Supplementary Fig. 2).

To show the structural advantages of TiO@C-HS as the sulfur host, four control samples are also prepared. First, TiO_2_@C-HS with a similar hollow structure is prepared by annealing PS@TiO_2_@PDA at 900 °C in the same atmosphere. The crystal phase of the product is rutile TiO_2_ (Fig. 3i; JCPDS No. 76–323). TiO_2_@C-HS also maintains uniform hollow spherical structure (Fig. 3j), while the grain sizes of the TiO_2_ nanocrystals are smaller than that of TiO@C-HS due to the lower annealing temperature (Fig. 3k). The lattice fringes of the nanocrystals are correlated to the (110) planes of rutile TiO_2_ (Fig. 3l). The carbon content of TiO_2_@C-HS is ∼40 wt% (Supplementary Fig. 3), which is lower than that of TiO@C-HS due to the relatively higher weight ratio of oxygen in the TiO_2_ shell. The second control sample is the carbon coated conductive TiO_2−*x*_ nanoparticles (TiO_2−*x*_@C-NP) synthesized by annealing the commercial TiO_2_ nanoparticles (Supplementary Fig. 4a,b) with PDA at 1,000 °C for 4 h in the same atmosphere of N_2_/H_2_ (95:5). In this sample, a mixture of TiO and Ti_4_O_7_ is obtained (Fig. 3m), and the carbon content is estimated to be 15–20 wt% (Supplementary Fig. 3). TEM and SEM images (Fig. 3n, Supplementary Fig. 4c,d) show abundant irregular nanoparticles with diameters from 40 to 150 nm, suggesting an obvious morphological evolution during the high temperature reduction treatment. The re-shaped TiO_2−*x*_ nanoparticles gain abundant exposed surface without being covered by carbon layers (Supplementary Fig. 5). High resolution TEM (HRTEM) observations further suggest the crystal phases of TiO (Fig. 3o) and Ti_4_O_7_ (Fig. 3p) nanoparticles in the TiO_2−*x*_@C-NP sample. Similar with the PS@TiO_2_ precursor, annealing of bare TiO_2_ nanoparticles yields very large particle sizes from 200 nm to 1 μm (Supplementary Fig. 4e,f), which once again proves that the carbon layer has an important role on controlling the particle size of titanium oxides during the high temperature treatment. To further demonstrate the effectiveness of the hollow structure and the important role of the polar TiO layer, commercial TiO_2_ nanoparticles (TiO_2_-NP) and pure carbon hollow spheres (C-HS) are applied as another two control groups. The TiO_2_-NP sample has mixed crystal phases of anatase and rutile (Fig. 3q) with particle sizes of 20–30 nm (Fig. 3r). The C-HS are synthesized by coating PS with PDA, followed by annealing at 900 °C in N_2_. The obtained C-HS exhibit an amorphous carbon phase (Fig. 3q) and well maintained hollow structure (Fig. 3s,t).

**Figure 4:**
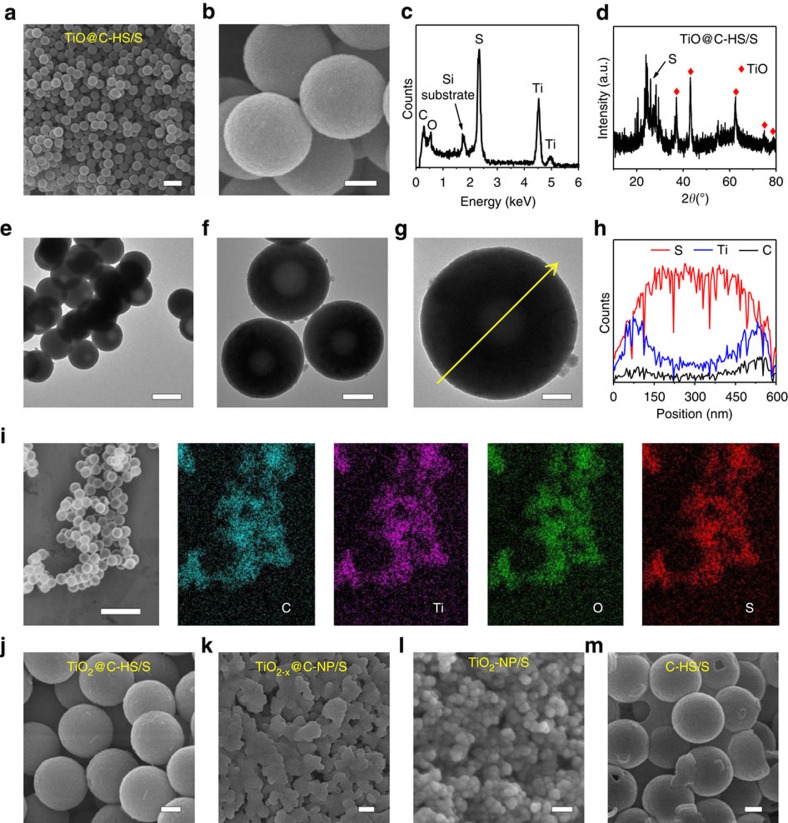
Characterization of the sulfur-based composite materials. (**a**,**b**) SEM image, (**c**) EDX spectrum, (**d**) X-ray diffraction pattern, (**e**–**g**) TEM images, (**h**) linear distributions of S, Ti and C along the arrow line on **g**, and (**i**) EDX mapping area and corresponding elemental distributions of C, Ti, O and S of TiO@C-HS/S. SEM images of (**j**) TiO_2_@C-HS/S, (**k**) TiO_2−*x*_@C-NP/S, (**l**) TiO_2_-NP/S and (**m**) C-HS/S. Scale bars, 1 μm (**a**), scale bars, 200 nm (**b**,**f**,**j**,**k**,**m**), scale bars, 500 nm (**e**), scale bars, 100 nm (**g**,**l**), scale bars, 2 μm (**i**).

Sulfur is loaded within the TiO@C-HS host by a modified vapour phase infusion method^50^. SEM observations reveal that the TiO@C-HS/S composite well maintains the original spherical shape with smooth surfaces (Fig. 4a,b), indicating that no extra sulfur exists outside the TiO@C-HS structure. Energy-dispersive X-ray (EDX) spectrum (Fig. 4c) and X-ray diffraction pattern (Fig. 4d) of the TiO@C-HS/S composite prove the presence of sulfur and TiO. TEM images (Fig. 4e,f) show that the contrast of the inner space of TiO@C-HS becomes much darker after sulfur loading, and the crystal nanoparticles of TiO on the shells cannot be easily identified. The linear scan analysis shows that the distribution of S is contrary to Ti and C (Fig. 4g,h), indicating that a high content of sulfur is present inside the TiO@C-HS host. The observations demonstrate that sulfur has been successfully accommodated and immobilized within the porous shells and the inner void spaces of TiO@C-HSs. EDX elemental mapping (Fig. 4i) of many TiO@C-HS/S nanospheres shows that sulfur is homogeneously distributed in the TiO@C-HS host. The TiO_2_@C-HS/S composite shows similar morphology with the TiO@C-HS/S (Fig. 4j), again suggesting the excellent sulfur loading capacity of hollow structures. However, for the TiO_2−*x*_@C-NP/S composite (Fig. 4k) and the TiO_2_-NP/S composite (Fig. 4l), thick layers of sulfur are evident on the surfaces of the host particles. After composing with sulfur by melt-diffusion at 155 °C, the C-HS/S composite still retains the original spherical morphology (Fig. 4m). To make a fair comparison, the sulfur contents in all five samples are controlled to be ∼70 wt% (Supplementary Fig. 6).

### Electrochemical performance

**Figure 5:**
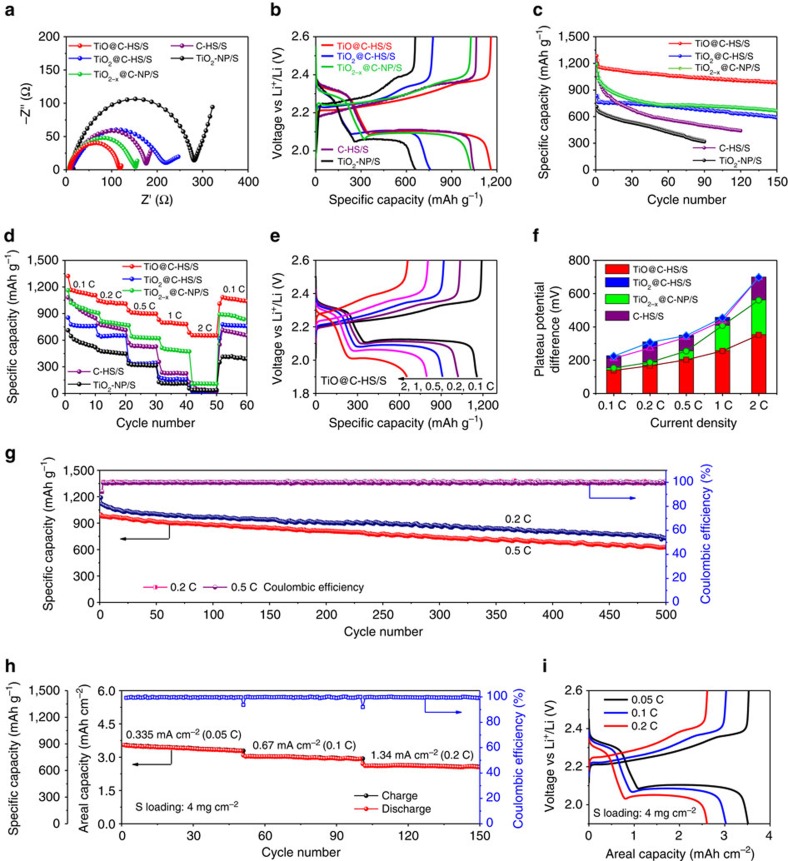
Electrochemical evaluation of TiO@C-HS/S. (**a**) Nyquist plots before cycling from 1 MHz to 100 mHz, (**b**) the second-cycle galvanostatic charge/discharge voltage profiles at 0.1 C, (**c**) cycle performances at 0.1 C, (**d**) rate capabilities and (**f**) the potential differences between the charge and discharge plateaus at various current densities of the TiO@C-HS/S, TiO_2_@C-HS/S, TiO_2−*x*_@C-NP/S, C-HS/S and TiO_2_-NP/S electrodes. (**e**) Voltage profiles at various current densities from 0.1 to 2 C and (**g**) prolonged cycle life and Coulombic efficiency at 0.2 and 0.5 C of the TiO@C-HS/S electrode. (**h**) Areal capacities and (**i**) voltage profiles at various current densities from 0.335 (0.05 C) to 1.34 mA cm^−2^ (0.2 C) of the TiO@C-HS/S electrode with high sulfur mass loading of 4.0 mg cm^−2^.

Both the sulfur mass loading and the amount of electrolyte injected in the coin cells are kept the same for all five samples in order to compare and evaluate the structural effects of host materials on the electrochemical properties. From the Nyquist plots (Fig. 5a), it can be observed that the TiO@C-HS/S electrode has the smallest semicircle in high-frequency region, indicating the lowest charge transfer resistance compared with TiO_2_@C-HS/S and TiO_2−*x*_@C-NP/S^43^. Since all cathodes contain approximately the same content of sulfur, the different charge transfer resistances could be attributed to the conductivity of the host materials. Benefitting from the metallic nature of TiO and the good confinement of sulfur, TiO@C-HS/S shows better ability to facilitate the charge transfer for surface reactions than the other sulfur hosts. On the contrary, due to the relatively poor conductivity of TiO_2_, the TiO_2_-NP/S electrode shows the highest charge transfer resistance. Figure 5b shows the second-cycle charge/discharge voltage profiles of the TiO@C-HS/S, TiO_2_@C-HS/S, TiO_2−*x*_@C-NP/S, C-HS/S and TiO_2_-NP/S electrodes at 0.1 C (1C=1,675 mA g^−1^). Among all samples, TiO@C-HS/S shows both highest first-discharge plateau at ∼2.33 V (corresponding to the reduction of sulfur to long-chain LiPSs) and longest second-discharge plateau at ∼2.1 V (the formation of short-chain LiPSs), suggesting much better redox reaction kinetics and more efficient utilization of the active sulfur material in TiO@C-HS/S. The cycling performance of five electrodes are compared at a relatively low current density of 0.1 C (Fig. 5c). Benefitting from the high conductivity of TiO, Ti_4_O_7_ and carbon, the electrodes of TiO@C-HS/S, TiO_2−*x*_@C-NP/S and C-HS/S deliver high initial discharge capacities of 1,285, 1,190 and 1,195 mAh g^−1^, respectively, while TiO_2_@C-HS/S and TiO_2_-NP/S only give 833 and 711 mAh g^−1^, respectively. It can be indicated that higher conductivity of the hosts promises better reaction kinetics of the active sulfur material. However, after 150 cycles, the discharge capacities of TiO_2−*x*_@C-NP/S drops to 664 mAh g^−1^, corresponding to capacity retention of only 56%. Another nanoparticle formed cathode of TiO_2_-NP/S delivers even worse cycling stability with the capacity retention of only 44% after 90 cycles. Without polar material in the host, C-HS/S shows the fastest fading ratio among all groups, and retains a capacity of only 442 mAh g^−1^ after 120 cycles, which is 37% of the initial capacity. In contrast, the TiO@C-HS/S and TiO_2_@C-HS/S electrodes show much better cycling stability with capacity retention of 76% and 71%, respectively, suggesting that hollow structured polar hosts can effectively block the diffusion of polysulfides and enhance the cycling stability.

Next, the rate capabilities and electrode kinetics of these cathode materials are evaluated at various current densities (Fig. 5d). When the current density is increased successively from 0.1C to 0.2, 0.5, 1 and 2C, the TiO@C-HS/S electrode delivers high stabilized specific capacities of 1,146, 1,029, 910, 800 and 655 mAh g^−1^, respectively (Fig. 5d,e). When the current density is reduced back to 0.1 C, the discharge capacity of TiO@C-HS/S is recovered to 1,083 mAh g^−1^, indicating good stability of the cathode structure after the high rate discharging and charging test. Compared with TiO@C-HS/S, both of the TiO_2−*x*_@C-NP/S and C-HS/S electrodes deliver obvious lower discharge capacities at various current densities from 0.1 C to 2 C (Fig. 5d, Supplementary Fig. 7a,b), which may be caused by the loss of active materials through LiPSs dissolution at the early cycles of the test. Although the TiO_2_@C-HS/S electrode also shows good cycling stability, it shows an abrupt capacity drop when the current density is increased to 0.5 C, and has almost no capacity at 2 C (Fig. 5d, Supplementary Fig. 7c). Because of the poor conductivity and ineffective LiPSs confinement of the bare TiO_2_ nanoparticles, the TiO_2_-NP/S electrode shows the worst rate capability among all groups (Fig. 5d). The calculated potential differences between the charge/discharge voltage plateaus at various current densities further confirm that the TiO@C-HS/S electrode possesses much less polarization and better reaction kinetics than the other samples (Fig. 5f), which can be attributed to its excellent conductivity and efficiency for LiPSs adsorption. The prolonged cycle life of the TiO@C-HS/S electrode is tested at 0.2 and 0.5 C for 500 cycles (Fig. 5g). After the initial discharge capacities of 1,190 and 1,066 mAh g^−1^, the TiO@C-HS/S electrode delivers capacities of 750 mAh g^−1^ at 0.2 C and 630 mAh g^−1^ at 0.5 C, respectively, corresponding to a small average capacity decay rate of ∼0.08% per cycle. The Coulombic efficiencies of the TiO@C-HS/S cells are >99% during the cycling process (Fig. 5g).

Since high mass loading of active materials and high areal capacities are essential for the energy density of Li-S batteries, a thick TiO@C-HS/S electrode with areal sulfur loading of 4.0 mg cm^−2^ is further evaluated (Fig. 5h,i). Upon cycling at 0.05 C, a discharge capacity of 886 mAh g^−1^ is delivered, corresponding to an areal capacity of 3.5 mAh cm^−2^. When the current density is increased to 0.1 and 0.2 C, the capacities are stabilized at above 730 mAh g^−1^ (2.9 mAh cm^−2^) and 630 mAh g^−1^ (2.5 mAh cm^−2^) over 50 cycles, respectively. The results of three different cells tested in the same condition demonstrate that the cells have good consistency, and the electrochemical performances are highly reproducible (Supplementary Fig. 8). The good cycling performance of a high mass-loading sulfur electrode requires good conductivity of the cathode materials, as well as efficient confinement of LiPSs. All these results show that TiO@C-HS is an attractive host material for the sulfur cathode of Li–S batteries to achieve stable cycling and high energy density. By comparing with many similar cathodes based on metal oxides/sulfides hosts (Supplementary Table 1 and Supplementary Fig. 9), it can be noted that the TiO@C-HS/S electrode of this work exhibits much enhanced areal capacities and very attractive cycling stability at high sulfur loading. Although the areal capacity reported here is not the highest compared with some recently reported carbon-based electrodes^51^,^52^,^53^, we believe that by further optimizing the design for the TiO/C-based cathode and other components in the cell, it is very possible to significantly enhance the electrochemical performance of Li–S battery.

## Discussion

**Figure 6:**
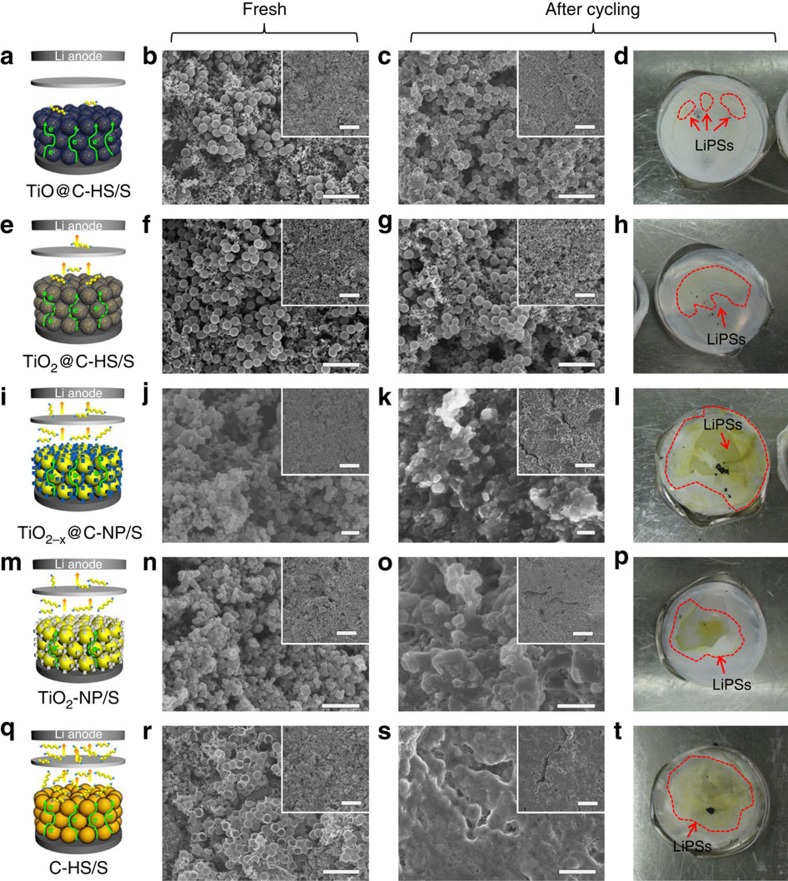
Characterization of electrode films and separators during cycling. (**a**,**e**,**i**,**m**,**q**) Schematic illustration of the mechanisms during redox reaction, SEM images of (**b**,**f**,**j**,**n**,**r**) fresh electrode films, (**c**,**g**,**k**,**o**,**s**) cycled electrode films and (**d**,**h**,**l**,**p**,**t**) digital photos of cycled separators of (**a**–**d**) TiO@C-HS/S, (**e**–**h**) TiO_2_@C-HS/S, (**i**–**l**) TiO_2−*x*_@C-NP/S, (**m**–**p**) TiO_2_-NP/S, and (**q**–**t**) C-HS/S. Scale bars, 2 μm (**b**,**c**,**f**,**g**,**r**,**s**), scale bars, 200 nm (**j**,**k**), scale bars, 500 nm (**n**,**o**), scale bars, 10 μm (insets of (**b**,**c**,**f**,**g**,**j**,**k**,**n**,**o**,**r**,**s**)).

The schematic illustrations of Li–S batteries show the different effects of various nanostructured sulfur hosts on electrochemical performance (Fig. 6a,e,i,m,q). The TiO@C-HS is the only structure that possesses both advantages of high conductivity and effective LiPSs confinement. To further demonstrate the superiority of TiO@C-HS for stable cycling performance, the electrode films and separators are examined after cycling. Cells are disassembled at fully charged status after cycling tests, and all of the electrode films and separators are directly used for the characterization without any treatment. SEM observations show that in the fresh electrodes, TiO@C-HS/S and TiO_2_@C-HS/S well maintain the spherical morphologies with unbroken shells (Fig. 6b,f), indicating good mechanical robustness of the hollow hosts for sustaining the electrode fabricating process. After cycling, the spherical TiO@C-HS/S and TiO_2_@C-HS/S composites show almost no variation compared with the pristine status (Fig. 6c,g), and the electrode films are also in good intact forms as the fresh ones (insets of Fig. 6b,c,f,g). As for TiO_2−*x*_@C-NP/S, TiO_2_-NP/S and C-HS/S, compared with the fresh electrodes (Fig. 6j,n,r), the expanded layers of LiPSs are all clearly evident on the surfaces of the cycled electrodes (Fig. 6k,o,s), and some cracks are generated on the electrode films (Inset of Fig. 6k,o,s). Since the separator is closely compressed on the surface of cathode electrode in the tightly sealed coin cell, the area of orange LiPSs on separator could also reflect their dissolution from the cathode. The trace of dissolved LiPSs on the separator from the TiO@C-HS/S cell shows inconspicuous colour and the smallest area, implying the most effective restriction of LiPSs in the TiO@C-HS/S cathode (Fig. 6d). In contrast, the orange areas on the separators of the TiO_2−*x*_@C-NP/S, TiO_2_-NP/S and C-HS/S cells are much larger, and the colours are more distinct than that of the TiO@C-HS/S and TiO_2_@C-HS/S cells (Fig. 6d,h,l,p,t), indicating that significant amount of LiPSs are dissolved from the TiO_2−*x*_@C-NP/S, TiO_2_-NP/S and C-HS/S cathodes during the cycling. These observations are in good agreement with the electrochemical test results, and visually demonstrate that TiO@C-HS serves as a great sulfur host for Li–S batteries.

**Figure 7:**
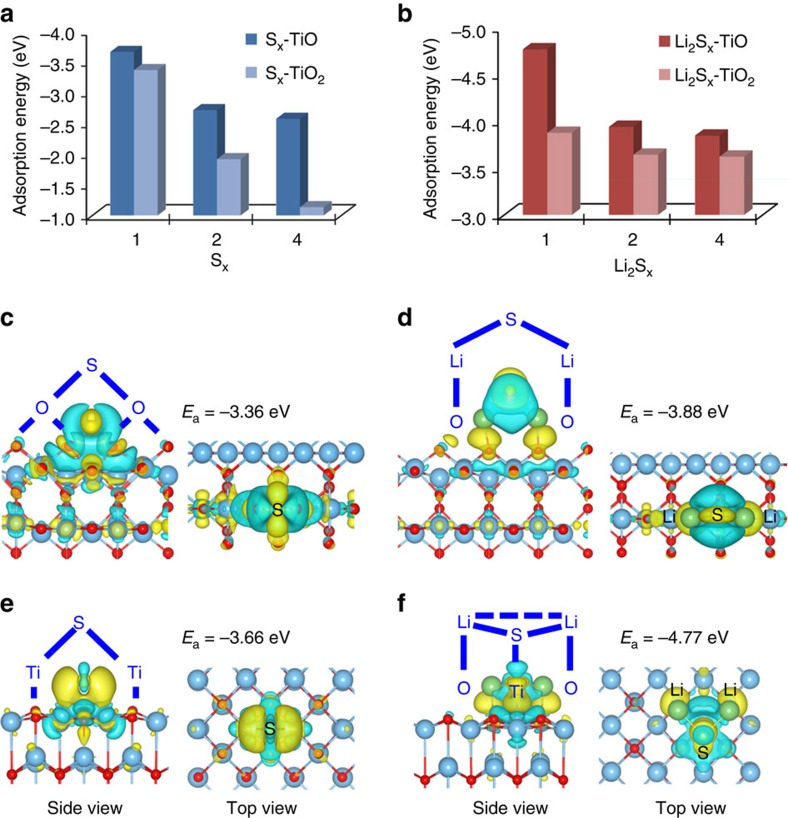
DFT analysis of adsorption energies of S species on TiO (001) and TiO_2_ (110). Adsorption energies for (**a**) S_*x*_ and (**b**) Li_2_S_*x*_ (*x*=1, 2 and 4) compounds on TiO (001) and TiO_2_ (110) surfaces. Isosurface of the charge density difference for (**c**) S and (**d**) Li_2_S adsorbed on TiO_2_ (110), and (**e**) S and (**f**) Li_2_S adsorbed on TiO (001). Yellow surfaces correspond to charge gains and blue surfaces correspond to an equivalent charge lost. To make the plot clear, the isovalues are defined as 0.002 in all cases.

To better reveal the superiority of TiO@C-HS on restricting the diffusion of LiPSs, the bonding properties of S_*x*_ and Li_2_S_*x*_ (*x*= 1, 2 and 4) on TiO (001) (ref. 54) and rutile TiO_2_ (110) (ref. 55) are studied by DFT calculations. The adsorption energy (*E*_a_) is calculated using the equation, *E*_a_=*E*_SS_+*E*_*X*_ (*X*=S, S_2_, S_4_, Li_2_S, Li_2_S_2_ and Li_2_S_4_)−E_total_. Here, *E*_total_, *E*_SS_ and *E*_*X*_ are the total energy of the whole system, substrate and molecular clusters, respectively, and the more negative *E*_a_ indicates the stronger adsorption capability. The calculated *E*_a_ of different adsorption systems are listed in Supplementary Table 2 and the optimized adsorption structures are shown in Supplementary Fig. 10. It is found that the S_*x*_/Li_2_S_*x*_ adsorptions on TiO (001) are much stronger than on TiO_2_ (110) (Fig. 7a,b and Supplementary Table 2). More importantly, the adsorption energies of S_2_ (−2.711 eV) and S_4_ (−2.571 eV) on TiO (001) are about 1 eV larger than the corresponding ones on TiO_2_ (110) (−1.914 eV for S_2_ and −1.134 eV for S_4_), thus TiO (001) plane has a striking ability to adsorb S_*x*_ clusters. The results indicate that, as the sulfur host, TiO can inhibit the dissolution of polysulfides clusters more effectively than rutile TiO_2_. To understand the underlying reason for the relative larger adsorption energies of both S_*x*_ and Li_2_S_*x*_ compounds on TiO (001), the electronic properties of the S_*x*_ and Li_2_S_*x*_ adsorbed on TiO (001) and TiO_2_ (110) systems are further analysed. The charge-differences show that the S atoms prefer to interact with the two surface oxygen atoms with the typical covalent bonds in S_*x*_/TiO_2_ (110) structures (Fig. 7c). This phenomenon could also be supported by other XPS and theoretical calculation results reported elsewhere^42^. Notably, with the extension of S chains (S→S_2_→S_4_), the chemical interaction between S_*x*_ and TiO_2_ (110) becomes weaker. In contrast, when S_*x*_ clusters adsorb on TiO (001), the S atoms strongly interact with the surface Ti_5c_ atoms with the character of the ionic interaction (Fig. 7e), which also leads to the more pronounced elongation of the S–S bonds in sulfur chains on TiO (001) than that on TiO_2_ (110) (Supplementary Table 2). These should be the main reason for the relatively larger adsorption energies of S_*x*_ on TiO (001). On the other hand, the strong interaction between Li_2_S_*x*_ and TiO (001) mainly originates from the large portion of the low coordinated Ti_5c_ active sites on TiO (001), since all Ti on the TiO (001) surface are Ti_5c_, while the ratio of Ti_5c_ and Ti_6c_ is 1:1 on the TiO_2_ (110) surface. Thus, the TiO host can provide stronger chemical adsorption energies for Li_2_S_x_ due to the formation of both Li–O and Ti–S bonds. Based on these DFT calculation results, it is reasonable to assume that the electrochemical performance of the TiO@C-HS/S composite originates from the unique surface chemical properties of TiO.

In summary, we have designed a sulfur host based on highly conductive polar TiO@C hollow nanospheres for lithium–sulfur batteries. This host can maximize the effectiveness of moderating LiPSs diffusion and enhance the redox reaction kinetics of sulfur species at the same time. Benefiting from the excellent conductivity and strong LiPSs adsorption capability of TiO@C shells, the TiO@C/S composite cathode delivers high discharge capacities of >1,100 mAh g^−1^ at 0.1 C, and exhibits stable cycle life up to 500 cycles at 0.2 and 0.5 C with a small capacity decay rate of 0.08% per cycle. In addition, when the areal loading of sulfur is increased to 4.0 mg cm^−2^, the TiO@C/S electrode can provide high areal capacities at various current densities with good stability and high Coulombic efficiency. This work overcomes the major limitations associated with other polar and nonpolar sulfur hosts, and may open up the prospect of constructing more efficient nanostructures for moderating the diffusion of LiPSs and enhancing the reaction kinetics of sulfur. Only with such high-efficiency sulfur cathodes, high-energy lithium–sulfur batteries can become possible in future.

## Methods

### Preparation of TiO@C-HS and other control host materials

PS nanospheres were prepared by a previously reported method^48^. TiO_2_ was firstly coated on PS via a method reported by our group^49^. Typically, ∼110 mg of PS was dispersed in 35 ml of ethanol, followed by adding 0.32 g of hexadecylamine and 0.8 ml of ammonium hydroxide. After stirring for 10 min, 0.2 ml of titanium isopropoxide was dropped into the dispersion under vigorous stirring. After reaction for 1 h, PS@TiO_2_ was collected by centrifugation and washed several times with ethanol and deionized water, and then dispersed in 150 ml of Tris-buffer aqueous solution (10 mmol l^−1^) under ultrasonication. After that, 80 mg of dopamine was added into the dispersion with magnetic stirring overnight. PS@TiO_2_@PDA was collected by centrifugation and washed several times with deionized water and ethanol, dried at 70 °C for 12 h. Finally, TiO@C-HS was prepared by annealing PS@TiO_2_@PDA in a reductive atmosphere of N_2_/H_2_ (95:5) mixture gas at 1,000 °C for 4 h with a heating rate of 5 °C min^−1^. TiO_2_@C-HS was obtained by annealing the same precursor of PS@TiO_2_@PDA at 900 °C for 2 h in the same atmosphere. For the synthesis of TiO_2−*x*_@C-NP, 100 mg of commercial TiO_2_ nanoparticles (Aeroxide P25, ACROS Organics) and 80 mg of dopamine were dispersed in 150 ml of Tris-buffer aqueous solution (10 mmol l^−1^) under ultrasonication, and then stirred for 12 h. The resultant TiO_2_/PDA composite was washed several times with deionized water and ethanol by centrifugation, and dried at 70 °C for 12 h. TiO_2−*x*_@C-NP was obtained by annealing TiO_2_/PDA at 1,000 °C for 4 h in the same atmosphere of N_2_/H_2_ (95:5). To prepare C-HS, 80 mg of PS and 160 mg of dopamine were dispersed into 150 ml of Tris-buffer solution (10 mmol l^−1^) with magnetic stirring overnight. The resultant PS@PDA was washed with water and ethanol for several times, and then dried at 70 °C for 12 h. The C-HS sample was obtained by annealing PS@PDA at 900 °C for 2 h in N_2_ atmosphere. TiO_2_-NP (Aeroxide P25) was heated at 110 °C for 12 h in oven before use.

### Preparation of TiO@C-HS/S and other control composites

The mixture of TiO@C-HS and sulfur powder (1:5, weight ratio) was sealed in a glass vessel under argon protection, and heated at 300 °C for 4 h in a quartz tube furnace for composing sulfur with TiO@C-HS. Then, the composite was placed in an open porcelain boat, and heated at 200 °C for 2 h in the quartz tube furnace under flowing argon gas to evaporate the extra sulfur that exists outside the TiO@C-HSs. After cooling down, TiO@C-HS/S was obtained. The preparation process of TiO_2_@C-HS/S is the same with the above mentioned method. Since the sulfur contents in both TiO@C-HS/S and TiO_2_@C-HS/S are around 70 wt%, the sulfur contents in TiO_2−*x*_@C-NP/S, C-HS/S and TiO_2_-NP/S are controlled to be about the same for a fair comparison. The TiO_2−*x*_@C-NP/S composite was prepared by heating the mixture of TiO_2−*x*_@C-NP and sulfur (3:7, w/w) at 155 °C for 12 h in a sealed glass vessel under argon protection. The C-HS/S and TiO_2_-NP/S composites were prepared by the same melt-diffusion method with TiO_2−*x*_@C-NP/S.

### Structural and phase characterization

The morphologies and structures of the samples were characterized by field-emission scanning electron microscope (JEOL-6700), TEM (JEOL, JEM-2010). Linear elemental scanning was recorded using EDX spectroscopy attached to TEM (JEOL, JEM-2100F). The crystal phases of the products were determined by a Bruker D2 Phaser X-Ray Diffractometer with Cu Kα radiation (*λ*=1.5406 Å). Sulfur or carbon contents of samples were determined by thermogravimetric analysis (Shimadzu DRG-60) in nitrogen or air flow, respectively.

### Electrochemical measurements

The electrode film was prepared by mixing 80 wt% of active material, 10 wt% of conductive carbon and 10 wt% of polyvinylidene fluoride in N-methylpyrrolidone, then the slurry was casted on the aluminium foil and dried at 70 °C overnight. The areal mass loading of sulfur is about 1.5 mg cm^−2^. The thick electrode film with sulfur loading of 4.0 mg cm^−2^ was composed of active material, conductive carbon and binder in the weight ratio of 70:20:10. The 2032-type coin cells were assembled using Celgard 2300 membrane as separator and Li metal as anode. The electrolyte was prepared by dissolving 1.0 mol l^−1^ lithium bis(trifluoromethanesulfonyl) imide in a mixture of 1,3-dioxolane and dimethoxymethane (1:1, v/v) with addition of 0.2 mol l^−1^ of LiNO_3_. The volume of electrolyte injected in coin cells is controlled as about 20 μl per 1 mg of electrode. The galvanostatic charge/discharge measurements were performed in a voltage range of 1.9–2.6 V using a NEWARE battery tester. The capacities were calculated based on the mass of sulfur. The electrochemical impedance measurements were carried out at 5 mV ac oscillation amplitude over the frequency range of 100 kHz to 100 MHz.

### Computational method

The calculations were performed using the Vienna Ab Initio Simulation Package in the framework of DFT^56^,^57^,^58^. The projector augmented wave pseudopotential was adopted and the generalized gradient approximation with the Perdew-Burke-Ernzerhof exchange-correlation (PBE) functional was used to treat the exchange-correlation interaction between electrons^59^. The cutoff energy of the projector augmented plane-wave basis set is 500 eV to ensure an accuracy of the energy of 1 meV per atom. The full geometry optimizations are carried out with the convergence thresholds of 10^−5^ eV and 1 × 10^−2^ eV Å^−1^ for total energy and ionic force, respectively. To get a reasonable description of the adsorption system, all the substrates are modelled with the periodic supercell. The *k*-space integration uses the Monkhorst-Pack scheme on a 2 × 2 × 1 and 1 × 2 × 2 mesh for TiO (001) and TiO_2_ (110) substrates, respectively. A 4 × 4 supercell slab of TiO containing 160 atoms (Ti_80_O_80_) was used. As for TiO_2_ (110), we employed a 2 × 4 supercell containing 192 atoms (Ti_64_O_128_) as the substrate.

### Data availability

The authors declare that the data supporting the findings of this study are available within the article and its Supplementary Information Files. All other relevant data supporting the findings of this study are available on request.

## Additional information

**How to cite this article**: Li, Z. *et al*. A sulfur host based on titanium monoxide@carbon hollow spheres for advanced lithium–sulfur batteries. *Nat. Commun.* **7**, 13065 doi: 10.1038/ncomms13065 (2016).

## Supplementary Material

Supplementary Information Supplementary Figures 1-10, Supplementary Tables 1-2 and Supplementary References

## Figures and Tables

**Figure 1:**
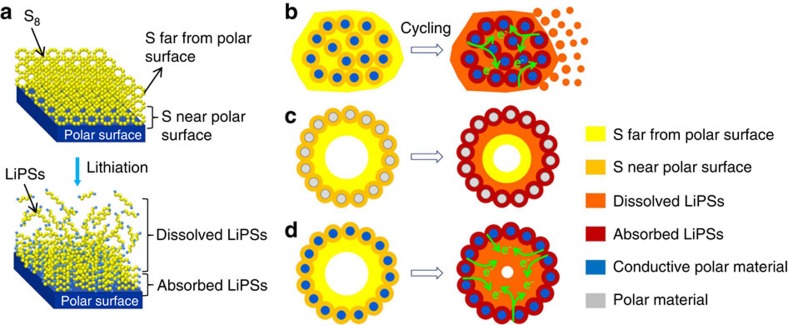
Schematic illustration of the adsorption limitation of LiPSs for polar hosts. (**a**) LiPSs can be chemically adsorbed only when they are close enough to the polar surface, LiPSs far from the polar surface cannot be effectively anchored during the cycling. (**b**) Conductive polar nanoparticles can chemically adsorb LiPSs near their surfaces. When the sulfur content of the composite exceeds the limit, the extra LiPSs would dissolve into the organic electrolyte. (**c**) The hollow polar structure can bond with LiPSs near the surface, and effectively restrict the diffusion of the inner LiPSs. However, the low conductivity of the host hinders high sulfur utilization. (**d**) The conductive polar hollow structure inherits both advantages of **b**,**c**.
