# Supplementary material for: A sulfur host based on titanium monoxide@carbon hollow spheres for advanced lithium–sulfur batteries
Source: Nat Commun. 2016 Oct 20;7:13065. doi: 10.1038/ncomms13065 (PMC5080434; doi:10.1038/ncomms13065)
Supplement: Supplementary Information — Supplementary Figures 1-10, Supplementary Tables 1-2 and Supplementary References [file ncomms13065-s1.pdf]

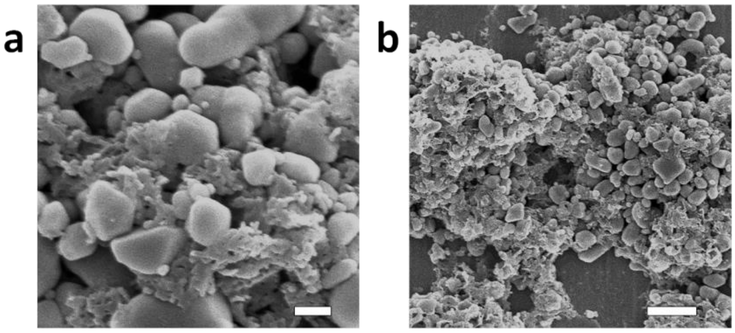

Supplementary Figure 1 | SEM images of the high-temperature annealed PS@TiO_2. (**a**, **b**) SEM images of the product prepared by annealing PS@TiO_2 at 1000 ℃ for 4 h in N_2/H_2 (95:5) atmosphere with a heating rate of 5 ℃ min^{-1}. Scale bars, 200 nm (**a**), 1 μm (**b**).

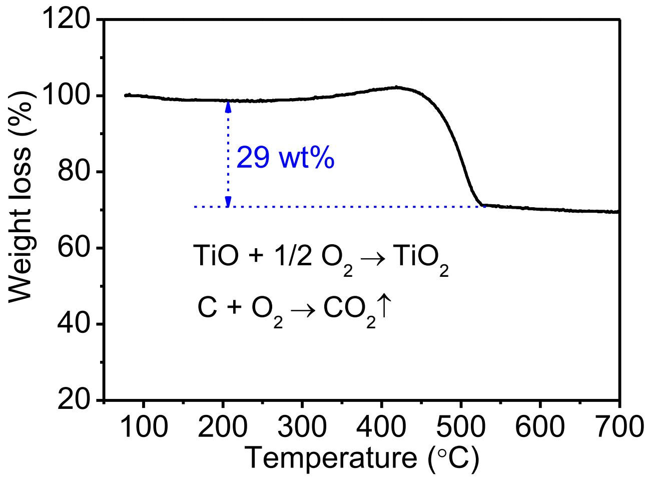

Supplementary Figure 2 | Thermogravimetric analysis of TiO@C-HS. TGA curve of TiO@C-HS in air atmosphere with a heating rate of 10 ℃ min^{-1}. Because the mass of TiO will increase 25 wt.% when it transforms into TiO_2 during the heating process in air, the weight loss of 29 wt.% of TiO@C-HS is the combined results of both weight increase of TiO and weight loss of carbon. The weight ratio of carbon (W_{carbon}) can be calculated by the following equation: $W_{carbon} – (100\% – W_{carbon}) \cdot 25\% = 29\%$, in which the weight ratio of TiO is ($100\% – W_{carbon}$). Therefore, the weight ratio of carbon is calculated as 43 wt.%, and then the weight ratio of TiO in TiO@C-HS is 57 wt.%.

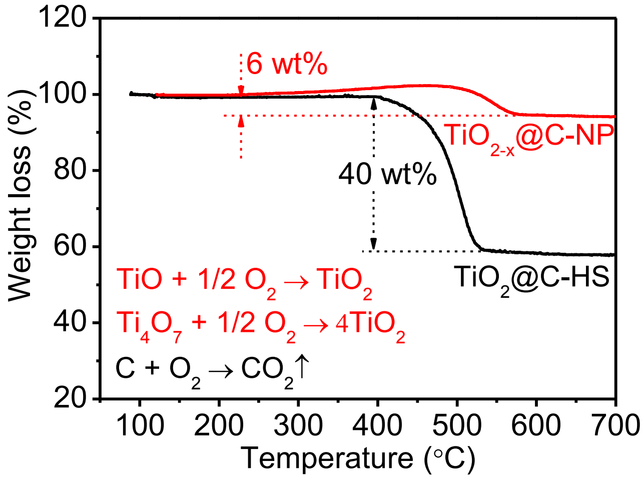

Supplementary Figure 3 | Thermogravimetric analysis of TiO_2@C-HS and TiO_{2-x}@C-NP. TGA curves of TiO_2@C-HS and TiO_{2-x}@C-NP in air atmosphere with a heating rate of 10 ℃ min^{-1}. The weight loss of 40 wt.% corresponds to the carbon content of the TiO_2@C-HS structure. When heated in air to 700 ℃, both TiO and Ti_4O_7 will transfer into TiO_2 phase, and the masses of TiO and Ti_4O_7 will increase 25 wt.% and 5.3 wt.%, respectively. Therefore, the 6 wt.% weight loss of TiO_{2-x}@C-NP resulted from the weight increase of TiO/Ti_4O_7, and the weight loss of carbon. If the weight increase only comes from TiO, the carbon content would be calculated as 24.8 wt.% by the equation of $W_{carbon} - (100\% - W_{carbon}) \cdot 25\% = 6\%$. On the other hand, when the weight increase only comes from Ti_4O_7, the carbon content would be 10.7 wt% based on the equation of $W_{carbon} - (100\% - W_{carbon}) \cdot 5.3\% = 6\%$. Since it is very difficult to determine the accurate weight ratio between TiO and Ti_4O_7 in the TiO_{2-x}@C-NP composite, the carbon content of TiO_{2-x}@C-NP can be speculated in the range of 10.7 - 24.8 wt.%. A good estimate will be in 15 – 20 wt.%.

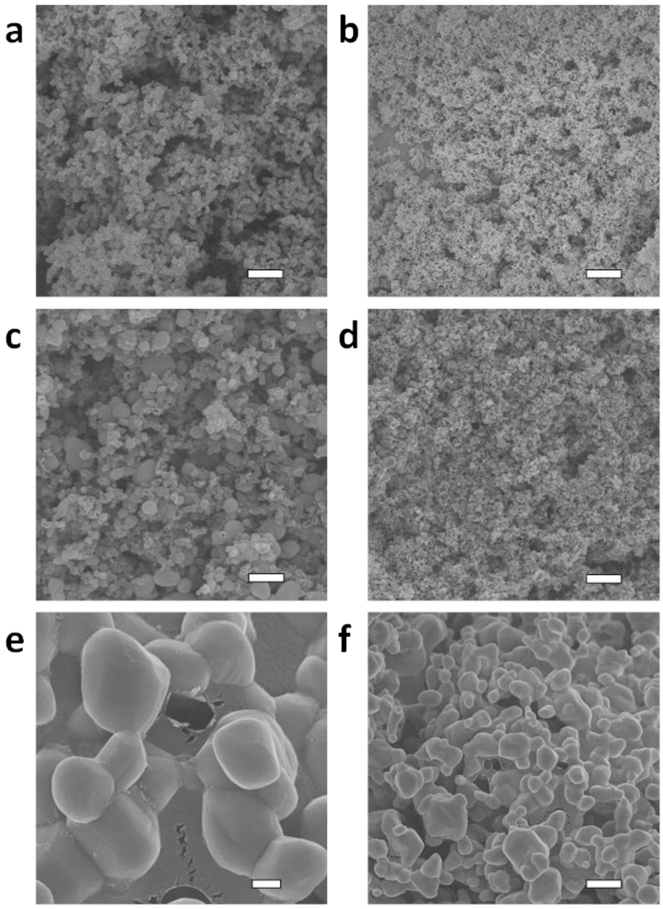

Supplementary Figure 4 | SEM characterizations of materials. SEM images of (**a, b**) commercial TiO_2 nanoparticles (TiO_2-NP) and (**c, d**) SEM images of TiO_{2-x}@C-NP. (**e, f**) the sample prepared by annealing bare TiO_2-NP at 1000 ℃ for 4 h in N_2/H_2 (95:5) atmosphere with a heating rate of 5 ℃ min^{-1}. Scale bars, 200 nm (**a,c,e**), 1 μm (**b,d,f**).

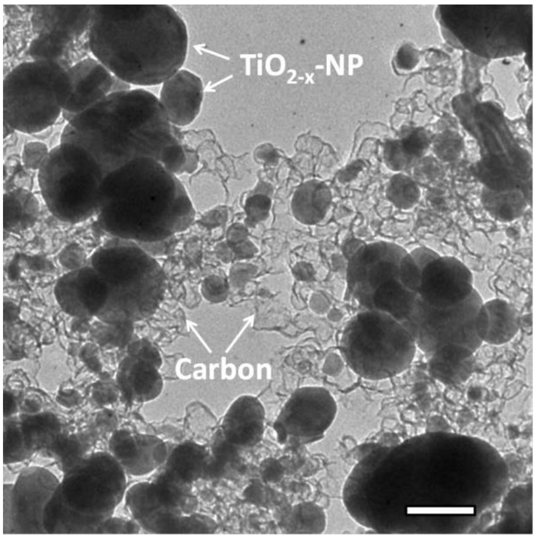

Supplementary Figure 5 | TEM image of TiO_{2-x}@C-NP. Most of TiO_{2-x} nanoparticles were recrystallized from the original TiO_2 nanoparticles after annealed at 1000 ℃ for 4 h, and the reshaped TiO_{2-x} nanoparticles show abundant exposed surface without being covered by carbon layers. Scale bar is 100 nm.

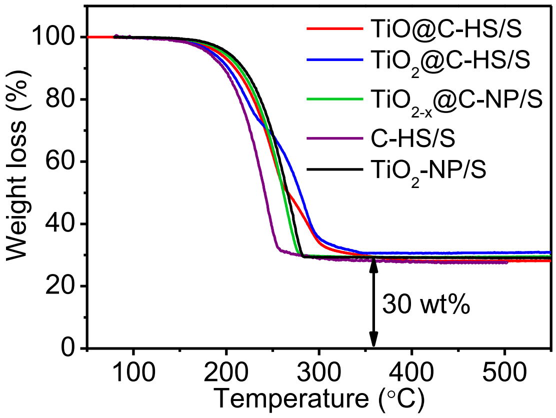

Supplementary Figure 6 | Thermogravimetric analysis of sulfur-based composites. TGA curves of TiO@C-HS/S, TiO_2@C-HS/S, TiO_{2-x}@C-NP/S, C-HS/S and TiO_2-NP/S in N_2 atmosphere with a heating rate of 10 ℃ min^{-1}.

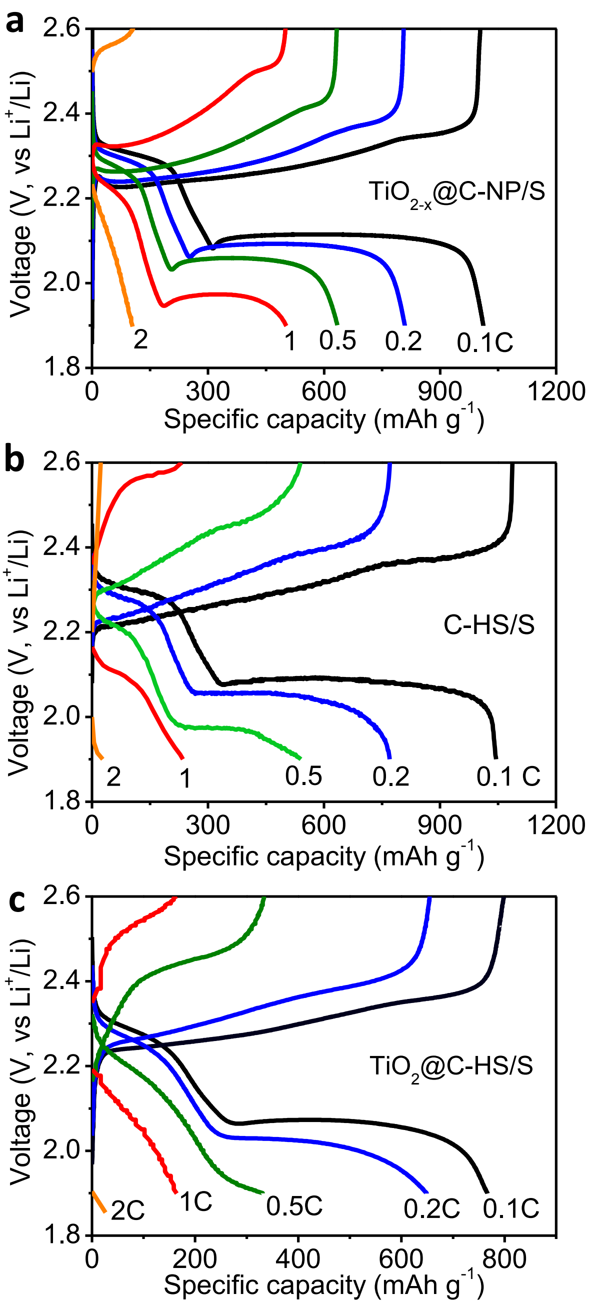

Supplementary Figure 7 | Voltage profiles comparison. Voltage profiles at various current densities from 0.1 to 2 C of the (**a**) TiO_{2-x}@C-NP/S, (**b**) C-HS/S and (**c**) TiO_2@C-HS/S electrodes.

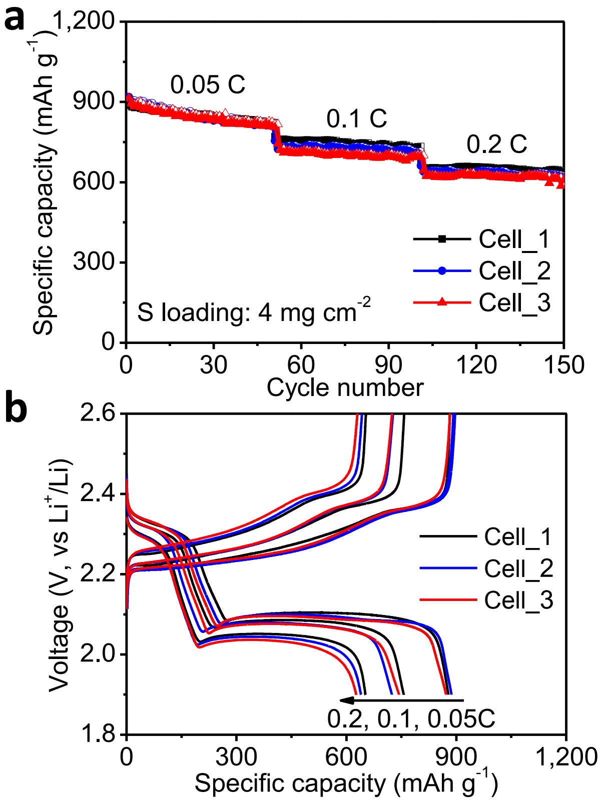

Supplementary Figure 8 | Reproducibility of the cell testing. (**a**) Cycling performance and (**b**) voltage profiles at various current densities of 3 different cells of TiO@C-HS/S with high sulfur mass loading of 4.0 mg cm^{-2} tested with the same experimental conditions.

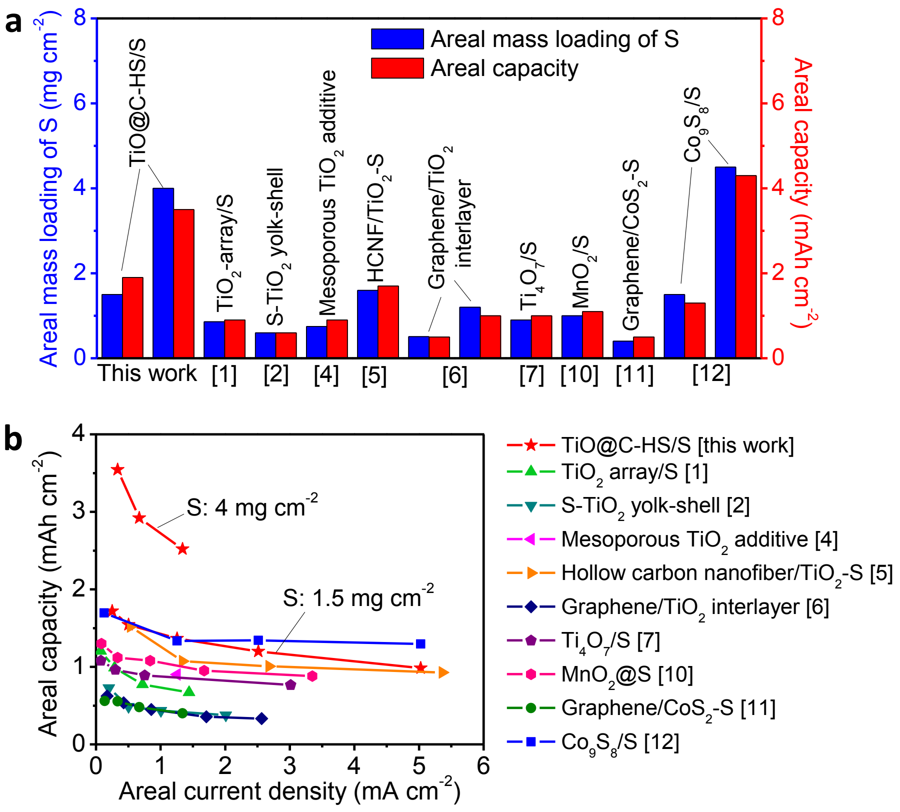

Supplementary Figure 9 | Electrochemical performance comparisons. (**a**) Areal capacities and (**b**) C-rate capacities comparisons of this work with some similar composite cathodes.

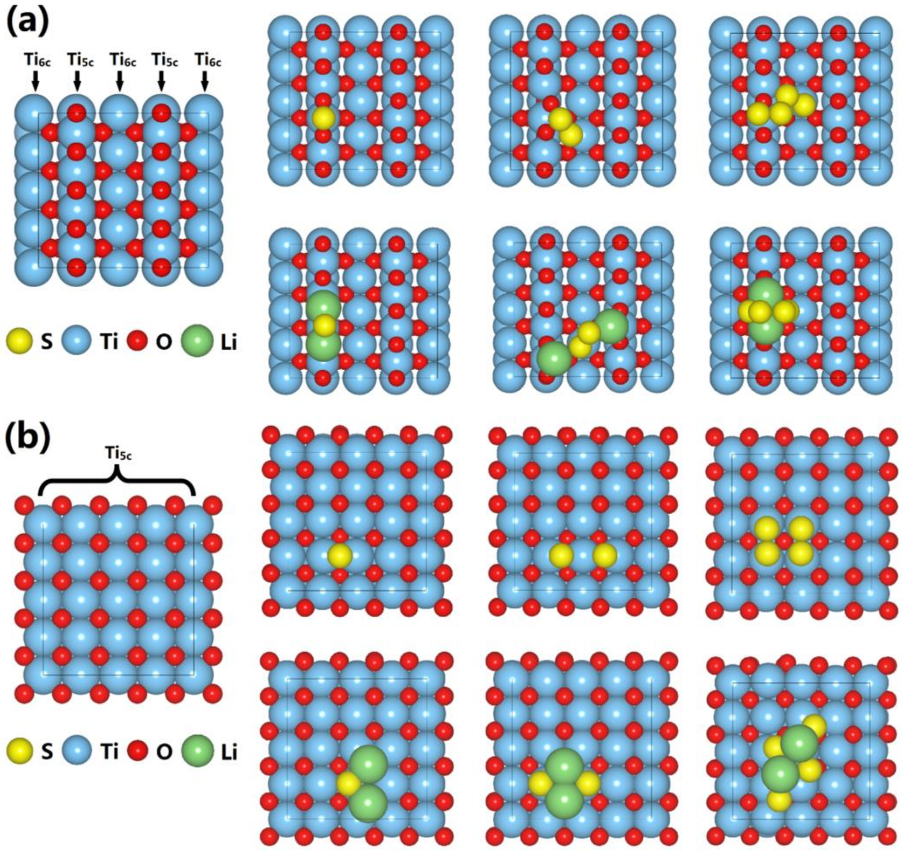

Supplementary Figure 10 | The optimized geometries for theoretical calculation. The optimized geometries for the interaction between S_x and Li_2S_x (x = 1, 2 and 4) on (**a**) TiO_2 (110) and (**b**) TiO (001) surfaces.

Supplementary Table 1. The performance comparison of this work with some similar composite cathodes.

Sample	S content	Areal mass loading of S (mg cm^{-2})	Initial cap. (mAh g^{-1})	Areal cap. (mAh cm^{-2})	Cycle stability* Finial cap. (mAh g^{-1})	Cycle stability* Cycles	Ref.
TiO@C-HS/S	70%	1.5	1285 at 0.1 C	1.9	750	500	**This work**
		4	886 at 0.05 C	3.5	821	50	
TiO_2-array/S	45%	0.86	1100 at 0.2C	0.9	890	200	**[1]**
S-TiO_2 yolk-shell	71%	0.4-0.6	1030 at 0.5C	0.6	690	1000	**[2]**
Mesoporous TiO_2/S	70%	NA	684 at 0.5 C	NA	676	100	**[3]**
Mesoporous TiO_2 additive	60%	0.75	1201 at 1 C	0.9	760	200	**[4]**
Hollow carbon nanofiber/TiO_2-S	67.5%	1.6	1040 at 0.5 C	1.7	650	200	**[5]**
Graphene/TiO_2 as interlayer	51%	0.51	1050 at 0.5 C	0.5	1040	300	**[6]**
	82%	1.2	802 at 0.5 C	1.0	600	250	
Ti_4O_7/S	60-70%	0.75-0.9	1000 at 0.5 C	1.0	800	250	**[7]**
Ti_4O_7/S	64.2%	NA	1342 at 0.02 C	NA	1034	100	**[8]**
Ti_2C/S	70%	NA	1090 at 0.5 C	NA	723	650	**[9]**
MnO_2/S	75%	0.7-1.0	1120 at 0.2 C	1.1	1030	200	**[10]**
graphene/CoS_2-S	75%	0.4	1368 at 0.5 C	0.5	1005	150	**[11]**
Co_9S_8/S	75%	1.5	890 at 0.5C	1.3	289	1500	**[12]**
	75%	4.5	956 at 0.05 C	4.3	~500	150	

*Cycle performances at moderate current densities are selected for the comparison.

Supplementary Table 2. The adsorption energies and the average S-S bond lengths of the most stable structures of S_x and Li_2S_x (x = 1, 2 and 4) species.

	TiO (001)		TiO_2 (110)	
	E_a (eV)	$l_{S\text{-}S}$ (Å)	E_a (eV)	$l_{S\text{-}S}$ (Å)
S	–3.657	\	–3.363	\
S_2	–2.7114	3.560	–1.914	1.943
S_4	–2.5705	2.354	–1.134	2.035
Li_2S	–4.7669	\	–3.876	\
Li_2S_2	–3.9395	\	–3.642	\
Li_2S_4	–3.8466	\	–3.622	\

Supplementary References

1. Liang, Z. et al. Sulfur cathodes with hydrogen reduced titanium dioxide inverse opal structure. *ACS Nano* **8**, 5249-56 (2014).

2. Seh, Z.W. et al. Sulphur-TiO_2 yolk-shell nanoarchitecture with internal void space for long-cycle lithium-sulphur batteries. *Nat. Commun.* **4**, 1331 (2013).

3. Ding, B., Shen, L.F., Xu, G.Y., Nie, P. & Zhang, X.G. Encapsulating sulfur into mesoporous TiO_2 host as a high performance cathode for lithium-sulfur battery. *Electrochim Acta* **107**, 78-84 (2013).

4. Evers, S., Yim, T. & Nazar, L.F. Understanding the nature of absorption/adsorption in nanoporous polysulfide sorbents for the Li–S battery. *J. Phys. Chem. C* **116**, 19653-8 (2012).

5. Zhang, Z. et al. Sulfur encapsulated in a TiO_2-anchored hollow carbon nanofiber hybrid nanostructure for lithium-sulfur batteries. *Chem. Eur. J.* **21**, 1343-9 (2015).

6. Xiao, Z. et al. A lightweight TiO_2/graphene interlayer, applied as a highly effective polysulfide absorbent for fast, long-life lithium-sulfur batteries. *Adv. Mater.* **27**, 2891-8 (2015).

7. Pang, Q., Kundu, D., Cuisinier, M. & Nazar, L.F. Surface-enhanced redox chemistry of polysulphides on a metallic and polar host for lithium-sulphur batteries. *Nat. Commun.* **5**, 4759 (2014).

8. Tao, X. et al. Strong sulfur binding with conducting magneli-phase Ti_nO_{2n-1} nanomaterials for improving lithium-sulfur batteries. *Nano Lett.* **14**, 5288-94 (2014).

9. Liang, X., Garsuch, A. & Nazar, L.F. Sulfur cathodes based on conductive mxene nanosheets for high-performance lithium-sulfur batteries. *Angew. Chem. Int. Ed.* **54**, 3907-11 (2015).

10. Liang, X. et al. A highly efficient polysulfide mediator for lithium-sulfur batteries. *Nat. Commun.* **6**, 5682 (2015).

11. Yuan, Z. et al. Powering lithium-sulfur battery performance by propelling polysulfide redox at sulfiphilic hosts. *Nano Lett.* **16**, 519-27 (2016).

12. Pang, Q., Kundu, D. & Nazar, L.F. A graphene-like metallic cathode host for long-life and high-loading lithium–sulfur batteries. *Mater. Horiz.* **3**, 130-6 (2016).
